# Polyaromatic Group Embedded Cd(II)-Coordination Polymers for Microwave-Assisted Solvent-Free Strecker-Type Cyanation of Acetals

**DOI:** 10.3390/molecules28030945

**Published:** 2023-01-18

**Authors:** Anirban Karmakar, Anup Paul, Maria Fátima C. Guedes da Silva, Armando J. L. Pombeiro

**Affiliations:** 1 Centro de Química Estrutural, Instituto Superior Técnico, Universidade de Lisboa, Av. Rovisco Pais, 1049-001 Lisbon, Portugal; 2 Research Institute of Chemistry, Peoples’ Friendship University of Russia (RUDN University), 6 Miklukho-Maklaya Street, 117198 Moscow, Russia

**Keywords:** coordination polymer, cyanation, acetal, crystal structure analysis, catalysis, cadmium

## Abstract

In this work, two new 1D Cd(II) coordination polymers (CPs), [Cd(L1)(NMF)_2_]_n_ (**1**) and [Cd(L2)(DMF)(H_2_O)_2_]_n_·n(H_2_O) (**2**), have been synthesized, characterized and employed as catalysts for the microwave-assisted solvent-free Strecker-type cyanation of different acetals. Solvothermal reaction between the pro-ligand, 5-{(pyren-1-ylmethyl)amino}isophthalic acid (**H_2_L1**) or 5-{(anthracen-9-ylmethyl)amino}isophthalic acid (**H_2_L2**), and Cd(NO_3_)_2_.6H_2_O in the presence of NMF or DMF:THF solvent, produces the coordination polymer **1** or **2**, respectively. These frameworks were characterized by single-crystal and powder X-ray diffraction analyses, ATR-FTIR, elemental and thermogravimetry analysis. Their structural analysis revealed that both CPs show one-dimensional structures, but CP **1** has a 1D double chain type structure whereas CP **2** is a simple one-dimensional network. In CP **1,** the dinuclear {Cd_2_(COO)_4_} unit acts as a secondary building unit (SBU) and the assembly of dinuclear SBUs with deprotonated ligand (L1^2−^) led to the formation of a 1D double chain framework. In contrast, no SBU was observed in CP **2**. To test the catalytic effectiveness of these 1D compounds, the solvent-free Strecker-type cyanation reactions of different acetals in presence of trimethylsilyl cyanide (TMSCN) was studied with CPs **1** and **2** as heterogenous catalysts. CP **1** displays a higher activity (yield 95%) compared to CP **2** (yield 84%) after the same reaction time. This is accounted for by the strong hydrogen bonding packing network in CP **2** that hampers the accessibility of the metal centers, and the presence of the dinuclear Cd(II) SBU in CP **1** which can promote the catalytic process in comparison with the mononuclear Cd(II) center in CP **2**. Moreover, the recyclability and heterogeneity of both CPs were tested, demonstrating that they can be recyclable for at least for four cycles without losing their structural integrity and catalytic activity.

## 1. Introduction

Coordination polymers (CPs) are highly promising 1D, 2D and 3D functional materials consisting of inorganic metal ions and organic linkers, which have attracted a high attention recently due to their interesting applications in gas storage and separation, heterogeneous catalysis, molecular separation, nonlinear optical, magnetism, sensing, photo, electrocatalysis, drug delivery, antitumor activity, bone and tumor therapy [1,2,3,4,5,6,7,8,9]. For the synthesis of functionalized coordination polymers, the selection of metal ions and the ligands with adequate binding centers is relevant [10,11]. Recently, a significant development in the synthesis of coordination polymers with polyaromatic groups bearing multidentate aromatic carboxylate ligands has been achieved with potential applications in various areas, such as gas storage, sensing and heterogenous catalysis [12,13]. However, the development of efficient synthetic strategies to obtain structures with predictable properties is still challenging.

On the other hand, numerous CPs have been studied as heterogeneous catalysts for various organic transformations, namely, cyanosilylation, Henry reaction, Knoevenagel condensations, transesterifications, tandem reactions, etc. [14,15,16,17,18], in view of their various advantages, such as insolubility, catalyst separation and recovery, and thermal stability, compared to homogeneous catalysts [19]. Moreover, CPs can catalyze any particular organic reaction either by their Lewis acidic metal centers or by acidic or basic functionalized ligands [19]. Thus, the synthesis of novel CPs containing functional organic linkers and their catalytic applications are a topic of potential interest [20,21].

In this context, the incorporation of the cyanide group in a molecule via C–C bond formation known as the cyanation reaction [22], is particularly interesting. Cyano compounds can be simply converted into various functional groups, e.g., aldehydes, amides and carboxylic acids, and, thus, are extensively used in pesticides, drugs, herbicides and pharmaceuticals industries [23]. In particular, the cyanation reaction of acetals is one of the important reactions in organic chemistry, which can produce various industrially valuable compounds [24]. Presently, several Lewis acid based homogenous catalysts, e.g., ZnI_2_, MgI_2_ etherate, SnCl_2_, BiBr_3_, TiCl_4_, etc. [25,26,27,28], as well as a few heterogenous coordination polymers [29], have been reported. However, they suffer from various limitations, such as the need of anhydrous conditions, high catalyst loading and inability for recycling. Hence, the synthesis of more resistant, less expensive and environmentally friendly heterogeneous catalysts for cyanation reaction of acetals is essential.

Moreover, the use of microwave (MW) irradiation in the organic transformations has emerged in recent years due to its potential advantages, namely, shorter reaction time and higher product yield and selectivity, compared to the traditional heating [30]. Moreover, there is an ever-growing need for an alternate energy source which will be cost effective as well as environmentally friendly and highly efficient. Various research groups including ours are employing this technique to perform numerous catalytic reactions under homo- and heterogenous conditions [31,32,33]. Even though catalytic activities of various CPs have been discovered under MW conditions [34,35], the microwave-assisted cyanation of acetals catalyzed by CPs has not yet been significantly studied. Thus, the discovery of suitable CP catalysts for the solvent-free microwave-assisted cyanation of acetals is a subject of potential attention.

Thus, in the present work, we have synthesized two novel 1D CPs, formulated as [Cd(L1)(NMF)_2_]_n_ (**1**) or [Cd(L2)(DMF)(H_2_O)_2_]_n_·n(H_2_O) (**2**) from the reactions of 5-[(pyren-1-ylmethyl)amino]isophthalic acid (**H_2_L1**) and 5-[(anthracen-9-ylmethyl)amino]isophthalic acid (**H_2_L2**) pro-ligands with Cd(NO_3_)_2_.6H_2_O, respectively. These CPs have been characterized by elemental, FT-IR, thermogravimetry single-crystal and powder X-ray diffraction analysis. Moreover, due to the presence of Lewis acidic Cd(II) metal center, we have tested their catalytic activity toward microwave-assisted solvent-free cyanation reactions of various acetals with trimethylsilyl cyanide (TMSCN). To our knowledge, this is the first example of a cyanation of acetals catalyzed by a coordination polymer under microwave irradiation. Finally, the heterogeneity and recyclability of these CPs were also investigated.

## 2. Results and Discussion

### 2.1. Synthesis and Characterization

**Scheme 1:**
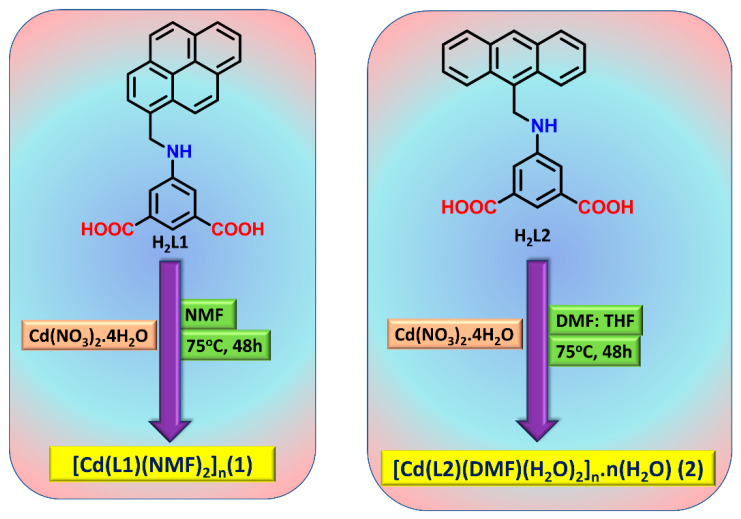
Synthesis of coordination polymers **1** and **2**.

The pro-ligands 5-{(pyren-1-ylmethyl)amino}isophthalic acid (**H_2_L1**) and 5-{(anthracene-9-ylmethyl)amino}isophthalic acid (**H_2_L2**) were synthesized by adopting previously reported procedures [8]. The solvothermal reaction of **H_2_L1** or **H_2_L2** with Cd(NO_3_)_2_.4H_2_O in the presence of N-methylformamide (NMF) or a dimethyl formamide (DMF) and tetrahydrofuran (DMF:THF) solvent mixture leads to the formation of the one-dimensional [Cd(L1)(NMF)_2_]_n_ (**1**) or [Cd(L2)(DMF)(H_2_O)_2_]_n_·n(H_2_O) (**2**) coordination polymer, respectively (Scheme 1).

In the FT-IR spectra of CP **1** and **2**, the characteristic asymmetric vibration band of coordinated carboxylate groups appears at 1537–1544 cm^−1^, whereas the symmetric vibration band occurs at 1368–1378 cm^−1^ [36]. The NH stretching vibration of the NH-groups are detected in the range of 3394–3420 cm^−1^. These CPs were also characterized by single crystal and powder X-ray diffraction analyses, elemental and thermogravimetry analysis.

Thermogravimetric analyses of CP **1** and **2** were executed in the range from 35 °C to ca. 800 °C under dinitrogen with a heating rate of 5 °C min^−1^. As shown in Figure S1 (Supplementary Materials), upon heating, CP **1** loses 19.1% of its original weight within temperature range of 85–258 °C, due to the elimination of two coordinated NMF molecules (calcd: 18.9%). Upon further rise in temperature the framework started to decompose slowly until 800 °C. CP **2** displays the weight loss of 8.6% within 53–158 °C due to the loss of one non-coordinated and two coordinated water molecules (calcd: 8.8%). Upon further heating, it exhibits another weight loss of 11.8% within temperature range of 159–254 °C, due to the loss of the coordinated DMF molecule (calcd: 12.0%). Afterwards, the framework starts to decompose.

### 2.2. Crystal Structure Analysis

**Figure 1:**
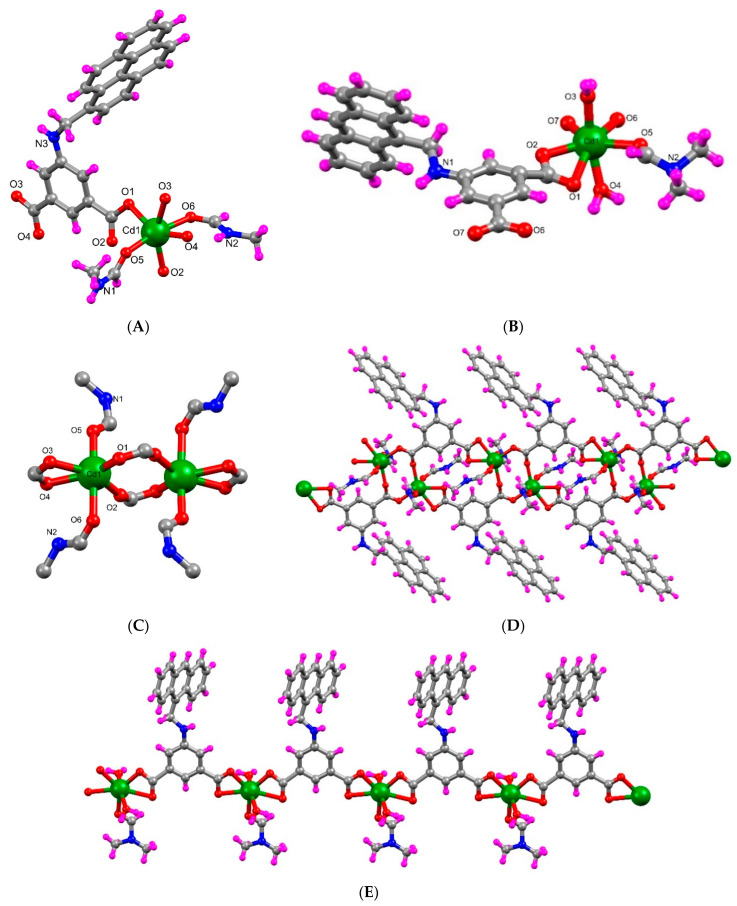
Asymmetric units of CP **1** (**A**) and CP **2** (**B**). (**C**) secondary building unit (SBU) of CP **1**. (**D**) One-dimensional double chain type structure of CP **1**. (**E**) One-dimensional chain of framework **2**.

Single-crystal X-ray diffraction studies revealed that CP **1** crystallizes in the triclinic P-1 space group, and the asymmetric unit contains one Cd(II) ion, one deprotonated L1^2−^ ligand and two coordinated N-methylformamide molecules (Figure 1A). It contains a one dimensional Cd(II) double chain. Each Cd(II) center presents a distorted octahedral coordination geometry and the four equatorial positions are occupied by four carboxylate oxygen atoms from three L1^2−^ ligands [Cd1-O1 2.237(2) Å, Cd1-O2 2.257(2) Å, Cd1-O3 2.367(2) Å, Cd1-O4 2.358(2) Å] and the remaining axial positions are coordinated by two N-methylformamide molecules [Cd1-O5 2.259(3) Å, Cd1-O6 2.371(3) Å]. In this framework, the organic ligand (L1^2−^) is not planar and the pyrene ring and the phenyl ring remain almost perpendicular to each other having a dihedral angle of 74.87°. One carboxylate group coordinates to the Cd(II) center in a monodentate fashion, whereas the other carboxylate groups show a bridging bidentate mode. The occurrence of a bulky pyrene ring in the ligand system inhibited the formation of a structure with a higher dimensionality, and a 1D double chain is formed (Figure 1D). Moreover, in this framework the dinuclear [Cd_2_(COO)_4_] unit acts as a secondary building unit (Figure 1C) and the distance between two symmetry related Cd(II) centers is 4.2042(4) Å. The combination of the dinuclear [Cd_2_(COO)_4_] unit along with the deprotonated L1 ligand leads to the construction of a one-dimensional double chain type framework as shown in Figure 1D.

**Figure 2:**
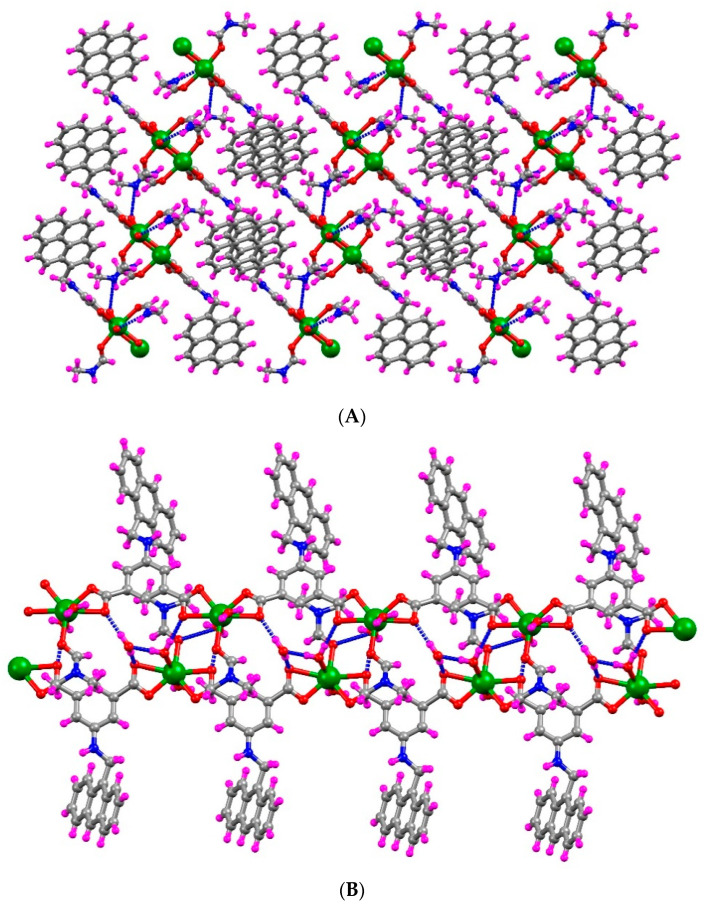
Two-dimensional hydrogen bonded structure of CP **1** (**A**) and one-dimensional hydrogen bonded network in CP **2** (**B**) (the hydrogen bonding interactions are indicated as blue dotted lines).

In this framework, the -NH groups of the coordinated N-methylformamide molecules are H-bonded via N1-H1N∙∙∙O1 (d_D–A_ 2.967(4) Å, D–H····A 151°) and N2-H2N∙∙∙O4 (d_D–A_ 2.880(4) Å, D–H····A 155°) interactions with a carboxylate-O atom and produce a 2D hydrogen-bonded network as shown in Figure 2A. Moreover, in this framework, the N-H····π (d_H····π_ 2.728 Å) interaction between the amine (-NH) group of the coordinated L1 ligand and the pyrene ring and the π····π interactions (d_π····π_ 3.363(6) Å) between the pyrene rings of two neighboring 1D chains are observed (Figure S2A). Moreover, different C-H····O interactions between C-H of NMF molecule and carboxylate-O also occur in this framework.

The asymmetric unit of the CP **2** contains one Cd(II) ion, one L2^2−^ ligand, one coordinated DMF, two coordinated and one lattice water molecules (Figure 1B). Each Cd(II) center displays a distorted hepta-coordinated pentagonal bipyramidal geometry with its equatorial positions being occupied by four COO^−^ oxygen atoms from two L2^2−^ units [Cd1-O1 2.665(2) Å, Cd1-O2 2.253(11) Å Cd1-O6 2.475(13) Å and Cd1-O7 2.313(12) Å] and one DMF molecule [Cd1-O5 2.354(14) Å], whereas the remining two axial positions are coordinated via two water molecules [Cd1-O3 2.260(2) Å and Cd1-O4 2.190(3) Å]. In this framework the combination of Cd(II) ion and deprotonated L2^2−^ ligand construct a 1D polymeric chain (Figure 1E). Like CP **1**, in CP **2** also the ligand L2^2−^ is non-planar due to the relative twisting of the CH_2_-NH group, and the pyrene and benzene ring of the ligand L2^2−^ are almost perpendicular having a dihedral angle of 64.49°. In this compound both the carboxylate groups are coordinated to the Cd(II) center in a chelating fashion. The Cd-Cd distance between two symmetry related Cd(II) ions in the 1D chain is 10.129(2) Å, which is considerably larger than the distance between two Cd(II) ions in vicinal chains (5.447(2) Å). In CP **2** the 1D chains are hydrogen bonded through various O-H····O interactions, such as O4-H4B∙∙∙O1S, O3-H3A∙∙∙O5, O3-H3B∙∙∙O4, O4-H4A∙∙∙O6, O1S-H1A∙∙∙O1 and O1S-H1B∙∙∙O1 (d_D–A_ 2.70(3)- 3.04(3) Å, D–H····A 139–179°), between the coordinated and non-coordinated water molecules, and between the coordinated water and the carboxylate-O atoms (Figure 2B). These interactions help to produce 2D hydrogen bonded networks which are more connected via C-H····π interactions between -CH of coordinated DMF and anthracene ring with a d_D-π_ distance of 3.013 Å (Figure S2A) and C-H····O interaction (d_D–A_ 3.309 Å) between anthracene CH and carboxylate-O atom, which expand the structure to the third dimension.

We have also performed topological analysis [37] of the hydrogen bonded networks of CPs **1** and **2**. The hydrogen bonded framework of CP **1** can be represented as a 2,6-connected binodal net (Figure S3A, Supporting Information) with topological type 2,6L1, whereas CP **2** exhibits a 3-connected uninodal net (Figure S3B, Supporting Information) with topological type hcb; Shubnikov hexagonal plane net.

### 2.3. Catalytic Study

**Scheme 2:**
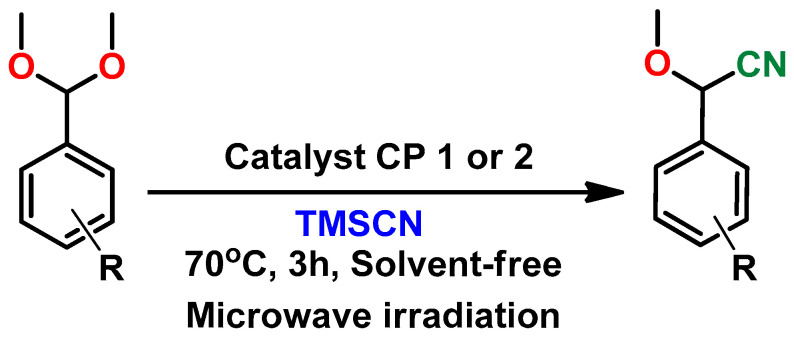
Schematic illustration of microwave assisted solvent-free cyanation of various acetals.

In the last few years, the use of microwave (MW) irradiation in catalysis has developed markedly due to its possible significant advantages, such as shorter reaction time and higher product yield, over traditional heating [30]. Thus, various research groups (including ours) performed different catalytic reactions by using MW irradiation [31,32,33,34,35]. Thus, we have also tested the catalytic activities of our newly synthesized CPs as heterogeneous catalysts for the Strecker-type cyanation reaction of various acetals with trimethylsilyl cyanide (TMSCN) in solvent-free conditions under microwave irradiation (Scheme 2).

In this typical reaction, a mixture of benzaldehyde dimethyl acetal (0.5 mmol), silylating agent trimethylsilyl cyanide (TMSCN) (1.0 mmol) and CP **1** or **2** as catalyst (0.5 to 3.0 mol %) was placed in a Pyrex tube covered with a Teflon cap and stirred at 25–90 °C under microwave irradiation (5 W) for 0.5–3 h under solvent-free conditions. After the desired reaction time, the reaction mixture was cooled and the catalyst was separated by centrifugation. The final product was determined through ^1^H NMR spectroscopy (Figure S6, Supporting Information) and the product yield was calculated using mesitylene as an internal standard as reported in the literature [29].

We have observed that CP **1** led to a higher product yield (95% after 3h) as compared to CP **2** (84% after 3 h), by using benzaldehyde dimethyl acetal and TMSCN as test compounds, under the same conditions. Thus, the experimental conditions have been optimized by changing the solvents, temperature (25–90 °C), catalyst loading (0.5–3 mol%) and reaction time, using CP **1** as the catalyst and the acquired results are shown in Table 1.

**Figure 3:**
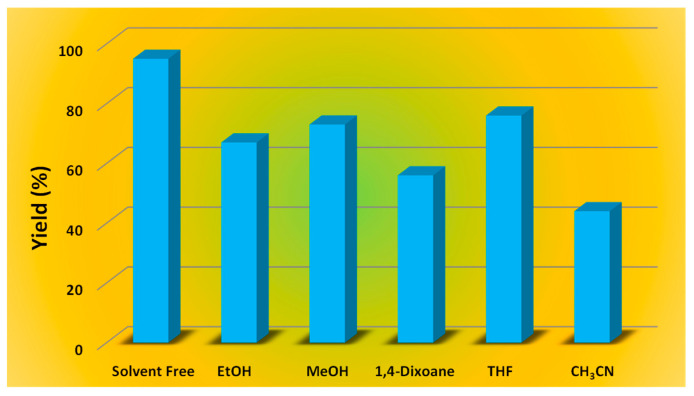
Yield of 2-methoxy-2-phenylacetonitrile in different solvents catalyzed by CP **1**.

In the optimization process, we have carried out the cyanation reactions in the presence of various solvents, such as EtOH, MeOH, 1,4-dioxane, THF or CH_3_CN, and under added solvent-free conditions (Figure 3). CPs **1** and **2** display the highest catalytic activity without any added solvent, with the product yield of 95% or 84%, respectively (entries 1 and 2, Table 1). By using THF as solvent and CP **1** as catalyst, benzaldehyde dimethyl acetal converts into 2-methoxy-2-phenylacetonitrile with a yield of 76% (entry 14, Table 1). The use of other solvents, such as EtOH, 1,4-dioxane, THF and CH_3_CN, leads to lower yields of 67, 73, 56 and 44%, respectively (entries 11–13, 15, Table 1).

The reaction temperature also plays a significant effect on the product yield. In fact, we have achieved only 30% and 77% of final product yield upon performing the reaction at 25 °C and 40 °C (entries 16 or 17, Table 1) using **1** as catalyst, respectively. However, increasing the reaction temperature from 40 °C to 70 °C results in a yield increase from 77% to 95% (entry 1, Table 1). Additional increase in the temperature to 90 °C has an opposite effect leading to the decrease in the reaction yield to 91% (entry 18, Table 1).

To optimize the catalyst loading for such a reaction, we have used 0.5, 1, 2 and 3 mol% of catalyst CP **1** under the above-mentioned experimental conditions. By using a lower amount of this catalyst (0.5 mol%) the final product yield of 71% was achieved (entry 19, Table 1), whereas increasing the catalyst load to 1 mol% positively increases the reaction yield to 95% (entry 1, Table 1). However, the product yields (94–95%, entries 20–21, Table 1) did not alter significantly upon an additional raise in the catalyst amount to 2 and 3 mol%. Thus, the above study reveals that 1 mol% of CP **1** is the optimal catalyst load for the reaction.

**Figure 4:**
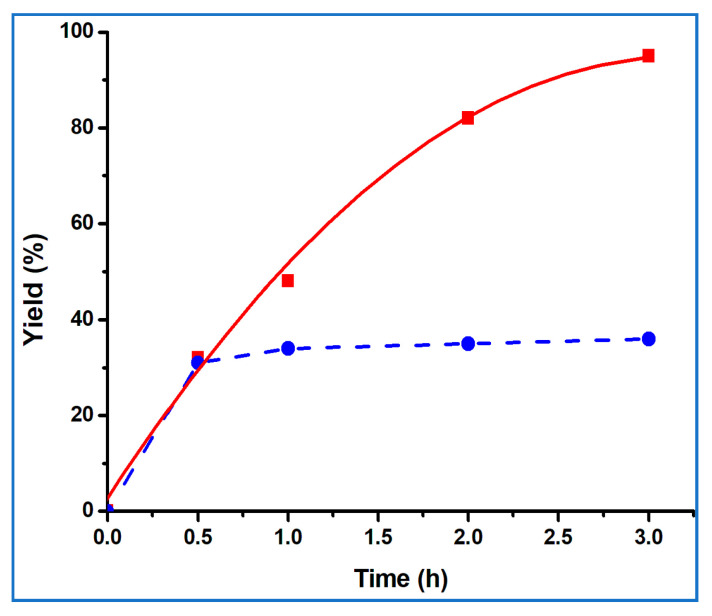
Yield vs. time plot of the microwave assisted solvent-free cyanation reaction between benzaldehyde dimethyl acetal and TMSCN catalyzed by CP **1** (red line, yield of 2-methoxy-2-phenylacetonitrile; dotted blue line, yield of this product with removal of the catalyst after 0.5 h of reaction time).

In order to find the optimal reaction time for the microwave assisted solvent-free cyanation reaction, we have monitored this reaction until 3 h at regular time intervals at 70 °C under solvent-free conditions in the presence of 1 mol% of catalyst CP **1** (optimized reaction conditions). A 32% product yield was achieved after 0.5 h of reaction time (entry 3, Table 1) and a continuing increase in the reaction time until 3 h led to an increase in yield to 95% (entries 4–6, Table 1). However, increasing the reaction time to 4 h did not improve the product yield. The yield vs. time plot for the catalyst CP **1** is represented in Figure 4.

For comparative purposes, we have also performed the solvent-free cyanation reaction under normal (conventional) heating conditions by using CP **1** catalyst at 70 °C for 3 h, which produced only 85% of final product yield (entries 7–10, Table 1). Thus, for our catalyst, the microwave irradiation method is more efficient in comparison with the conventional heating conditions.

We have also performed the microwave assisted cyanation reaction without any catalyst (blank reaction) which led to a yield of only 5% after 3 h (entry 23, Table 1) at 70 °C. The use of the metal salt Cd(NO_3_)_2_.4H_2_O, as well as the free ligands (H_2_L1 and H_2_L2), led to the higher yields (in comparison with the blank) of 43% and 7–10% (entries 22, 24 and 25, Table 1), respectively, suggesting that the Cd(II) ion has a greater role compared to the ligands in such a reaction.

**Figure 5:**
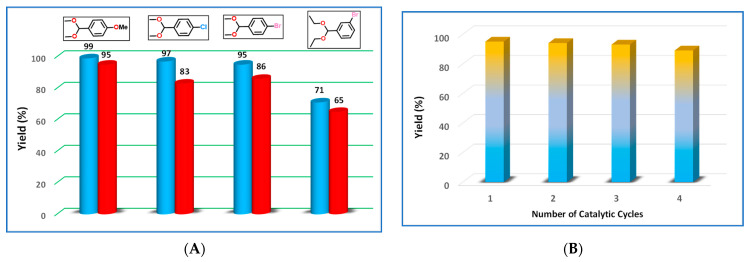
(**A**) Yield of product (2-methoxy-2-phenylacetonitrile) for the microwave assisted solvent-free cyanation reaction of different substituted acetals with trimethylsilyl cyanide (TMSCN) catalyzed by CP **1** [cyan pillar] and CP **2** [red pillar]. (**B**) Effect of catalyst recycling on the product yield for the microwave assisted solvent-free cyanation reaction between benzaldehyde dimethyl acetal and TMSCN catalyzed by CP **1**.

**Scheme 3:**
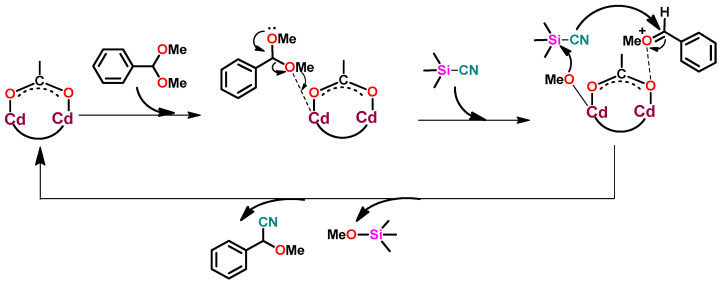
Proposed reaction mechanism for cyanation of acetals with TMSCN in presence of our catalyst CP **1**.

The microwave assisted cyanation reaction was also undertaken with different substituted benzaldehyde dimethyl and diethyl acetals and it was observed that an electron-donating substituent (4-methoxy) produces a higher yield (yields 95–99%) in comparison to an electron-withdrawing substituent (4-bromo or 4-chloro) (yields 83–97%) for both the catalysts CP **1** and **2** (entries 26–33, Table 1) (Figure 5A). The electron donor effect of the *para*-methoxy group at the phenyl ring increases the electron density at the C-atom of the CH(OMe)_2_ group which subsequently helps the release of the methoxide anion and generation of an oxocarbenium cation and promote the catalytic reaction [38] (see Scheme 3 below). By using diethyl acetal (3-bromo benzaldehyde diethyl acetal) instead of dimethyl acetal, much lower final product yields around 65–71% were achieved (entries 32–33, Table 1) under optimal reaction conditions.

These studies also disclosed that CP **1** displays a higher catalytic activity than CP **2** under optimal reaction conditions. This is consistent with the expected steric effect of the tight H-bonding packing in CP **2** that hampers the reaction. However, it can also be accounted for the presence of a dinuclear Cd(II) SBU in CP **1** which can promote the catalytic process (as suggested by the proposed reaction mechanism in Scheme 3) in comparison with the mononuclear Cd(II) center present in CP **2**.

Catalysis leaching and recycling concern important aspects to be considered in heterogeneous catalytic processes. To check if catalyst CP **1** leaching occurs, we performed a hot filtration test experiment as described by Sheldon et al. [39]. In this process, we stirred the reaction mixture for 0.5 h at 70 °C and then the catalyst was removed by centrifugation. Afterwards, the catalyst-free solution was kept under microwave irradiation for another 2.5 h. The product yield did not increase significantly as shown in Figure 4 (blue dotted line) after removal of the catalyst CP **1**, which evidences the heterogeneous nature of our catalyst. Moreover, we also determined that only 0.015% of Cd(II) ions were present in the solution after removal of the catalyst from the reaction mixture, which also indicates that almost no leaching happened during the catalytic reaction.

Furthermore, to check the reusability of our catalyst CP **1**, we reused it successively at least for four times, and no significant decrease into its catalytic efficiency was observed (Figure 5B). Moreover, the structural integrity of our CPs before and after the cyanation reaction were examined by FT-IR and powder X-ray diffraction (PXRD) analyses (Figures S4 and S5) and no significant changes were observed. Thus, we can conclude that the catalyst’s structure is preserved.

However, as far as we are aware, examples of microwave assisted cyanation reaction of acetals are not found in the literature; we compare, herein, the activity of various Lewis acid/bases homo- and heterogenous catalysts with our newly synthesized CPs **1** and **2** towards the cyanation of benzaldehyde dimethyl acetal (Table S1, Supporting Information). For example, by using a Lewis acid salt such as BiBr_3_ or MgI_2_ etherate as catalyst and TMSCN as cyanating agent, 89 and 53% yield of 2-methoxy-2-phenylacetonitrile was reached within 1 h and 36 h of reaction time, respectively (entries 1–2, Table S1) [26,28]. Moreover, some other Lewis acid salts, e.g., TiCl_4_ and SiCl_3_OTf, in the presence of different cyanating agents such as *t*-butyl isocyanide and cyanoamine, led to 95–96% yields within 0.5 or 3 h of reaction time at −70 or 0 °C, respectively (entries 5 and 6, Table S1) [38,40]. The use of other types of catalysts such as tetracyanoethylene and triethylsilyl trifluoromethanesulfonate (TESOTf) led to 72% and 98% of yields after 5 h reaction time, respectively (entries 3 and 4, Table S1), which is a higher reaction time in comparison with our catalytic systems [41,42]. Examples of heterogenous catalysts for such a reaction are rare. Recently we have reported two Cd(II) based CPs which efficiently catalyzed the cyanation reaction of acetals with 94–96% yields that are reached after 4 h at 80 °C (entries 7–8, Table S1) [29] which concern higher reaction time and temperature compared to our CPs **1** and **2** catalysts (entries 9–10, Table S1).

Inspired by the literature [38], we propose the catalytic mechanism in Scheme 3 for the cyanation of acetal catalyzed by CP **1**. In this proposed mechanism, first, an oxocarbenium cation species and a methoxide ligand are formed through the activation of the acetal by a dinuclear Cd(II) center. This is followed by the transfer of the CN^−^ group from TMSCN to the oxocarbenium cation yielding the cyanated product 2-methoxy-2-phenylacetonitrile by C-C bond formation and the elimination of (CH_3_)_3_SiOMe as the by-product. A related mechanism can be suggested for CP **2**, although involving a mononuclear Cd(II) center.

## 3. Experimental Section

Syntheses of 5-((pyren-1-ylmethyl)amino)isophthalic Acid (H_2_L1) and 5-{(anthracen-9-ylmethyl)amino)isophthalic Acid} (H_2_L2) were performed according to previously reported methods.

### 3.1. Synthesis of [Cd(L1)(NMF)_2_]_n_ (1)

An equimolar mixture of H_2_L1 (10.0 mg, 0.025 mmol) and Cd(NO_3_)_2_.4H_2_O (7.7 mg, 0.025 mmol) was placed in a 5 mL capped glass vessel and dissolved in 2 mL of N-methylformamide. The capped glass vial was heated at 75 °C for 48 h. After cooling the reaction mixture to room temperature (0.5 °C min^−1^) colorless crystals of **1** were obtained. Yield: 56% (based on H_2_L1). Anal. Calcd. for C_29_H_25_CdN_3_O_6_ (*M* = 623.94): Calculated: C, 55.82; H, 4.04; N, 6.73; Found: C, 55.48; H, 4.11; N, 6.36. FT-IR (cm^−1^): 3420 (w), 3209 (wb), 1670 (s), 1647 (s), 1599 (w), 1544 (s), 1413 (s), 1368 (s), 1331 (m), 1148 (w), 1037 (m), 1016 (m), 988 (w), 931 (w), 872 (s), 800 (s), 752 (s), 729 (w).

### 3.2. Synthesis of [Cd(L2)(DMF)(H_2_O)_2_]_n_·n(H_2_O) (2)

A 3:1 mixture of Cd(NO_3_)_2_.4H_2_O (24.9 mg, 0.081 mmol) and H_2_L2 (10.0 mg, 0.027 mmol) was placed in a 5 mL glass vessel and dissolved in a 2 mL mixture of DMF:THF (1:2 *v*/*v*). Subsequently, the reaction mixture was closed and heated for 48 h at 75 °C. Slow cooling to room temperature (0.3 °C min^−1^) provided colorless crystals. Yield: 43% yield (based on H_2_L2). Anal. Calcd. for C_26_H_28_CdN_2_O_8_ (*M* = 608.90): Calculated: C, 51.28; H, 4.63; N, 4.60; Found: C, 51.41; H, 4.36; N, 4.39. FT-IR (cm^−1^): 3394 (w), 1641 (s), 1537 (s), 1494 (m), 1420 (s), 1373 (s), 1320 (m), 1282 (m), 1168 (w), 1138 (w), 1110 (w), 1090 (w), 998 (m), 936 (s), 906 (s), 778 (m), 733 (s), 693 (s).

### 3.3. Procedure for the Microwave Assisted Solvent-Free Cyanation of Acetals

Benzaldehyde dimethyl acetal (0.076 g, 0.50 mmol), trimethylsilyl cyanide (125 µL, 1.0 mmol) and 1.0 mol% catalyst (3.1 mg of **1** or 3.0 mg of **2**, with respect to the acetal) was mixed and placed in a Pyrex tube. The Pyrex tube was then placed in the microwave reactor and irradiated with stirring under MW irradiation (15 W) at 70 °C under solvent-free conditions for 0.5–3 h. After cooling the reaction mixture, the catalyst was then separated by centrifugation and the yield of final crude product was calculated by ^1^H NMR in CDCl_3_ using the internal standard method. A representative example of ^1^H-NMR spectra is shown in Figure S6 (Supporting Information).

## 4. Conclusions

In conclusion, we have synthesized and characterized two novel Cd(II) coordination polymers formulated as [Cd(L1)(NMF)_2_]_n_ (**1**) and [Cd(L2)(DMF)(H_2_O)_2_]_n_·n(H_2_O) (**2**). The single crystal X-ray diffraction analysis shows that the CP **1** has a 1D double chain type structure, whereas CP **2** is a simple one-dimensional network. Moreover, in CP **1** dinuclear {Cd_2_(COO)_4_} units act as SBUs but, in contrast, no SBU formation was observed in CP **2**. We also observed different types of hydrogen bonding interactions in both frameworks. These interactions resulted in the generation of a three-dimensional hydrogen bonded network.

The insolubility in organic solvents and the existence of Lewis acidic Cd(II) centers in these CPs make them suitable heterogeneous Lewis acid based catalysts for the microwave assisted solvent-free cyanation reactions of acetals. To our knowledge, this is the first reported example of microwave assisted cyanation reaction of acetals utilizing coordination polymers as catalysts. Under optimized reaction condition CP **1** displays a higher activity (95% after 3 h at 70 °C) compared to CP **2** (84% after 3 h at 70 °C), possibly due to the presence of a dinuclear Cd(II) SBU in CP **1** which helps to promote the catalytic process. For comparative purposes, we have also performed the solvent-free cyanation reaction under conventional heating conditions, and the obtained lower product yield proves the effectiveness of the microwave irradiation method in comparison to the normal heating, toward such a reaction. Moreover, we have also tested the recyclability and heterogeneity of both CPs, showing that they can be recyclable at least for four cycles without losing structural integrity (checked by PXRD and FT-IR analyses) or catalytic activity, thus also proving their stability under the conditions employed.

The cyanation of acetals is a field that remains underdeveloped, and further investigation on the synthesis of novel heterogenous catalysts for such a reaction deserves to be pursued. Thus, this work contributes to a significant addition on the design of new heterogeneous CP based catalysts for cyanation reactions.

## Figures and Tables

**Table 1:** Microwave assisted cyanation of benzaldehyde dimethyl acetal with trimethylsilyl cyanide (TMSCN) in the presence of catalyst CP 1 or 2 ^a^.

Entry	Catalyst	Catalyst Amount (mol%)	Temp (°C)	Microwave Frequency (W)	Solvent	Time (h)	Yield (%) ^b^
1	**1**	1	70	15	No solvent	3	95
2	**2**	1	70	15	No solvent	3	84
3	**1**	1	70	15	No solvent	0.5	32
4	**1**	1	70	15	No solvent	1	48
5	**1**	1	70	15	No solvent	2	82
6	**1**	1	70	15	No solvent	3	95
7 ^c^	**1**	1	70	-	No solvent	0.5	25
8 ^c^	**1**	1	70	-	No solvent	1	39
9 ^c^	**1**	1	70	-	No solvent	2	72
10 ^c^	**1**	1	70	-	No solvent	3	84
11	**1**	1	70	15	EtOH	3	67
12	**1**	1	70	15	MeOH	3	73
13	**1**	1	70	15	1,4-Dioxane	3	56
14	**1**	1	70	15	THF	3	76
15	**1**	1	70	15	CH_3_CN	3	44
16	**1**	1	25	15	No solvent	3	30
17	**1**	1	40	15	No solvent	3	77
18	**1**	1	90	15	No solvent	3	91
19	**1**	0.5	70	15	No solvent	3	71
20	**1**	2	70	15	No solvent	3	94
21	**1**	3	70	15	No solvent	3	95
22	Cd(NO_3_)_2_.4H_2_O	1	70	15	Solvent free	3	43
23	Blank	-	70	15	Solvent free	3	5
24	**H_2_L1**	-	70	15	Solvent free	3	7
25	**H_2_L2**	-	70	15	Solvent free	3	10
26 ^d^	**1**	1	70	15	No solvent	3	>99
27 ^d^	**2**	1	70	15	No solvent	3	95
28 ^e^	**1**	1	70	15	No solvent	3	97
29 ^e^	**2**	1	70	15	No solvent	3	83
30 ^f^	**1**	1	70	15	No solvent	3	95
31 ^f^	**2**	1	70	15	No solvent	3	86
32 ^g^	**1**	1	70	15	No solvent	3	71
33 ^g^	**2**	1	70	15	No solvent	3	65

^a^ Reaction conditions: benzaldehyde dimethyl acetal (75 µL, 0.5 mmol) and trimethylsilyl cyanide (125 µL, 1 mmol), catalyst **1** or **2** (0.5–3 mol %), temperature (25–90 °C), time (0.5–3 h) and microwave irradiation (15 W). ^b^ Calculated by ^1^H-NMR as the number of moles of final product 2-methoxy-2-phenylacetonitrile per mole of benzaldehyde dimethyl acetal X 100. ^c^ Reaction performed under normal heating conditions. ^d^ 4-Methoxybenzaldehyde dimethyl acetal as substrate. ^e^ 4-Chlorobenzaldehyde dimethyl acetal as substrate. ^f^ 4-Bromobenzaldehyde dimethyl acetal as substrate. ^g^ 3-Bromobenzaldehyde diethyl acetal as substrate.

## Data Availability

The data presented in this study are available on request from the corresponding authors.

## Supplementary Materials

The following supporting information can be downloaded at: https://www.mdpi.com/article/10.3390/molecules28030945/s1. Figure S1: Thermogravimetric analysis curves of CPs 1 and 2; Figure S2: (A) N-H····π and π····π interactions in coordination polymer 1. (B) C-H····π and C-H····O interactions in coordination polymer 2; Figure S3: Topological representation of hydrogen bonded networks of CP 1 and CP 2 (the metal nodes are represented in pink and the linkers in green color); Figure S4: FT-IR spectra of CP 1 (A) and 2 (B) before (red) and after (blue) the cyanation of acetal; Figure S5: PXRD spectra of CP 1 (A) and 2 (B) simulate (black), as synthesized (red) and after (green) the cyanation reaction; Figure S6 (A) 1H-NMR spectra of the crude product of the solvent-free cyanation of benzaldehyde dimethyl acetal with CP 1 as catalyst in CDCl3 (entries 1 and 2, Table 1) (The protons are considered in the integrations are indicated in red colour). (B) Time depended (0.5h to 3h) 1H-NMR spectrum for the cyanation of benzaldehyde dimethyl acetal with catalyst CP 1; Table S1: A comparison of catalytic activity of various catalysts in the cyanation of benzaldehyde dimethyl acetal reactions; Table S2: Crystal data and structure refinement details for compounds 1-2; Table S3: Hydrogen bond geometry (Å, °) in compounds 1-2; Table S4: Selected bond distances (Å) and angles (°) for compounds 1–2.
