# Supplementary material for: Polyaromatic Group Embedded Cd(II)-Coordination Polymers for Microwave-Assisted Solvent-Free Strecker-Type Cyanation of Acetals
Source: Molecules. 2023 Jan 18;28(3):945. doi: 10.3390/molecules28030945 (PMC9919643; doi:10.3390/molecules28030945)
Supplement: Supplementary file 1 [file molecules-28-00945-s001.zip › molecules-2116415-supplementary.pdf]

Supplementary Material

Polyaromatic Group Embedded Cd(II)-Coordination Polymers for Microwave-assisted Solvent-free Strecker-type Cyanation of Acetals

Anirban Karmakar [1,*], Anup Paul [1], Maria Fátima C. Guedes da Silva [1] and Armando J.L. Pombeiro [1,2,*]

[1] Centro de Química Estrutural, Instituto Superior Técnico, Universidade de Lisboa, Av. Rovisco Pais, 1049–001 Lisbon, Portugal
[2] Peoples' Friendship University of Russia (RUDN University), Research Institute of Chemistry, 6 Miklukho-Maklaya Street, Moscow 117198, Russia
* Correspondence: anirban.karmakar@tecnico.ulisboa.pt (A.K.); pombeiro@tecnico.ulisboa.pt (A.J.L.P.)

1. Materials and Methods

All the chemicals were obtained from commercial sources and used without further purification. For the ligands synthesis and catalysis, the following chemicals from Sigma-Aldrich were used: 1-pyrenecarboxaldehyde (purity of ≥ 99%), 9-anthracenecarboxaldehyde (purity of ≥ 97%), 5-aminoisophthalic acid (purity of ≥ 94%), and benzaldehyde dimethyl acetal (purity of ≥ 99%), trimethylsilyl cyanide (purity of ≥ 98%). For the CP synthesis, $Cd(NO_3)_2.4H_2O$ (purity of ≥ 99%) from Acros Organics and three different solvents, namely dimethylformamide (DMF) (purity ≥ 99.5%), THF (purity ≥ 99.8%), N-methylformamide (purity ≥ 99.8%) from Fisher Scientific, were used. The synthesis of ligands and coordination polymers was performed in air and at relatively high temperatures. The infrared spectra (4000–400 cm^{-1}) were recorded on a Agilent Cary 630 ATFT-IR spectrometer; abbreviations: s = strong, m = medium, w = weak, bs = broad and strong, mb = medium and broad. ^1H (300 MHz) NMR spectra were obtained at room temperature (RT) on a Bruker Avance II + 300 (UltraShieldTMMagnet) spectrometer. CHN elemental analyses were carried out by the Microanalytical Service of the Instituto Superior Técnico. Thermogravimetric analyses were carried out in the 30-800 °C range at a heating rate of 5 °C min^{-1} under a dinitrogen atmosphere with a Perkin-Elmer Instrument system (STA6000). Powder X-ray diffraction (PXRD) was conducted in a D8 Advance Bruker AXS (Bragg Brentano geometry) θ-2θ diffractometer, with copper radiation (Cu Kα, λ = 1.5406 Å) and a secondary monochromator, between 5° and 40° operated at 40 kV and 40 mA.

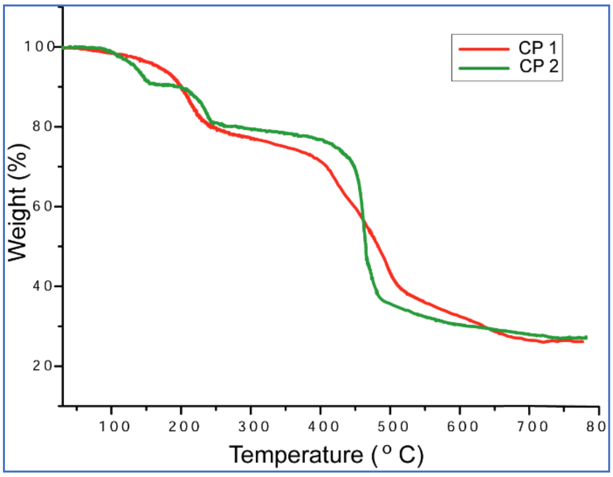

Figure S1. Thermogravimetric analysis curves of CPs **1** and **2**.

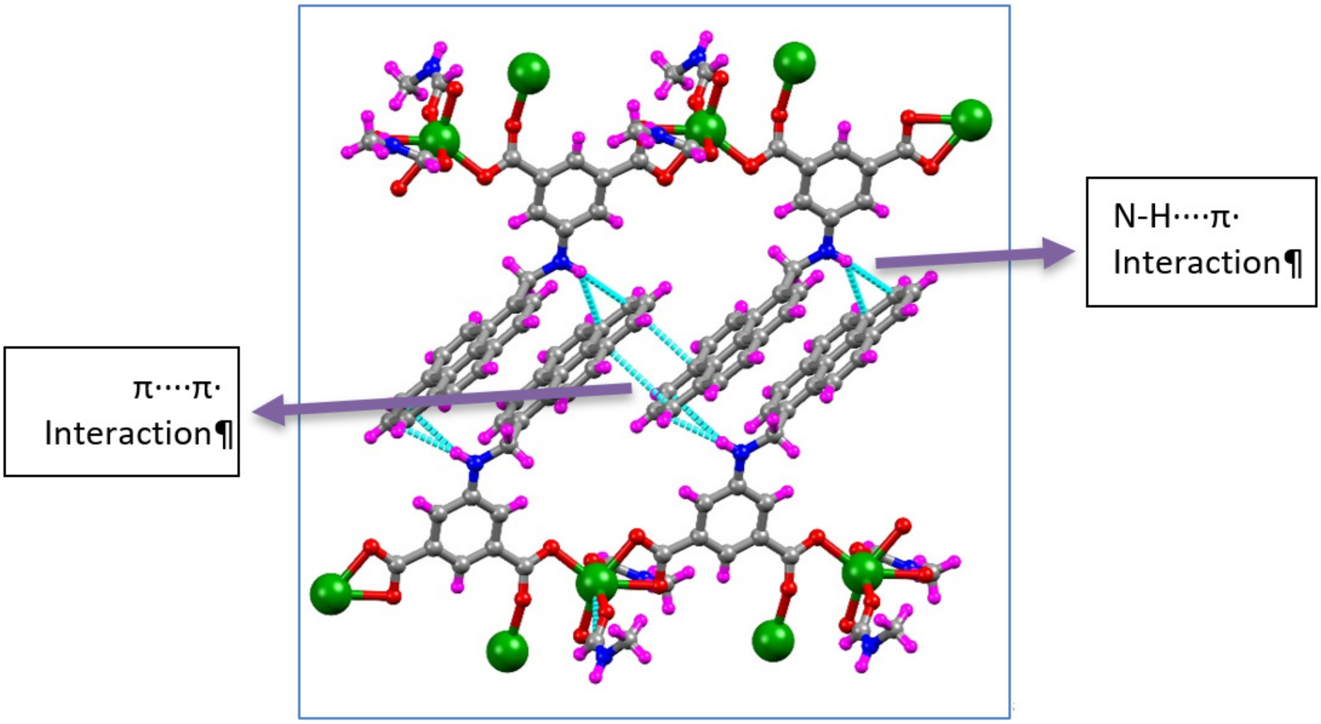

(A)

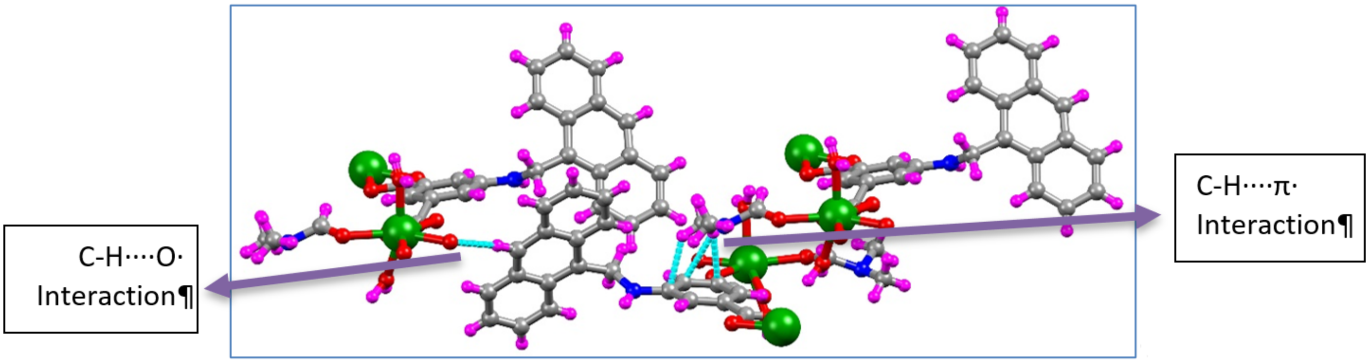

(B)

Figure S2. (A) N-H····π and π····π interactions in coordination polymer **1**. (B) C-H····π and C-H····O interactions in coordination polymer **2**.

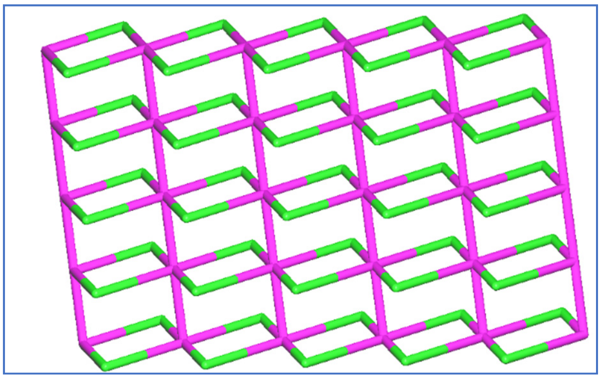

(A)

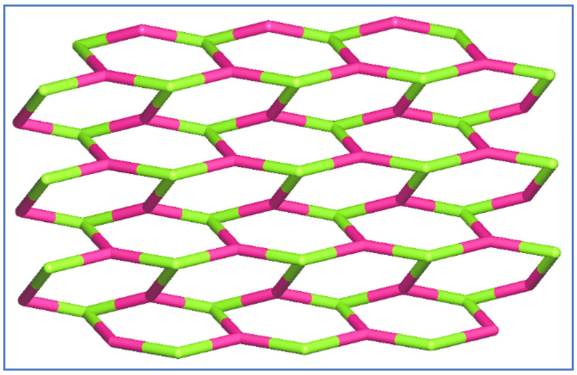

(B)

Figure S3. Topological representation of hydrogen bonded networks of CP **1** and CP **2** (the metal nodes are represented in pink and the linkers in green color).

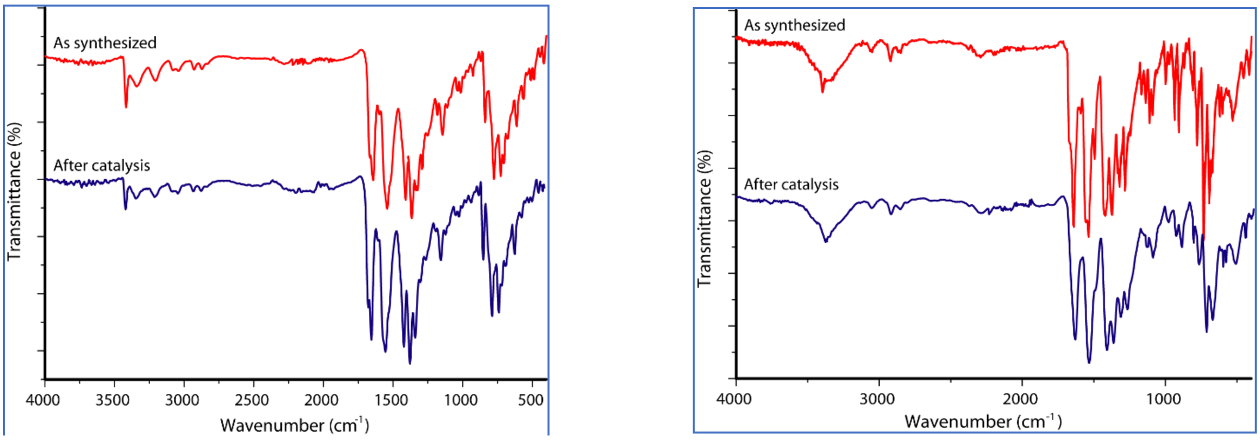

(A) (B)

Figure S4. FT-IR spectra of CP **1** (**A**) and **2** (**B**) before (red) and after (blue) the cyanation of acetal.

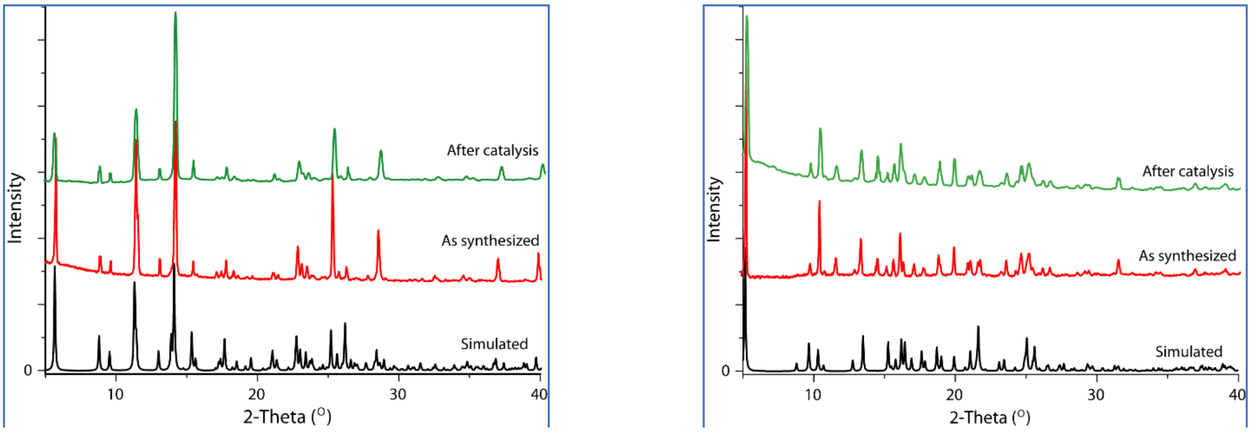

(A) (B)

Figure S5. PXRD spectra of CP **1** (**A**) and **2** (**B**) simulate (black), as synthesized (red) and after (green) the cyanation reaction.

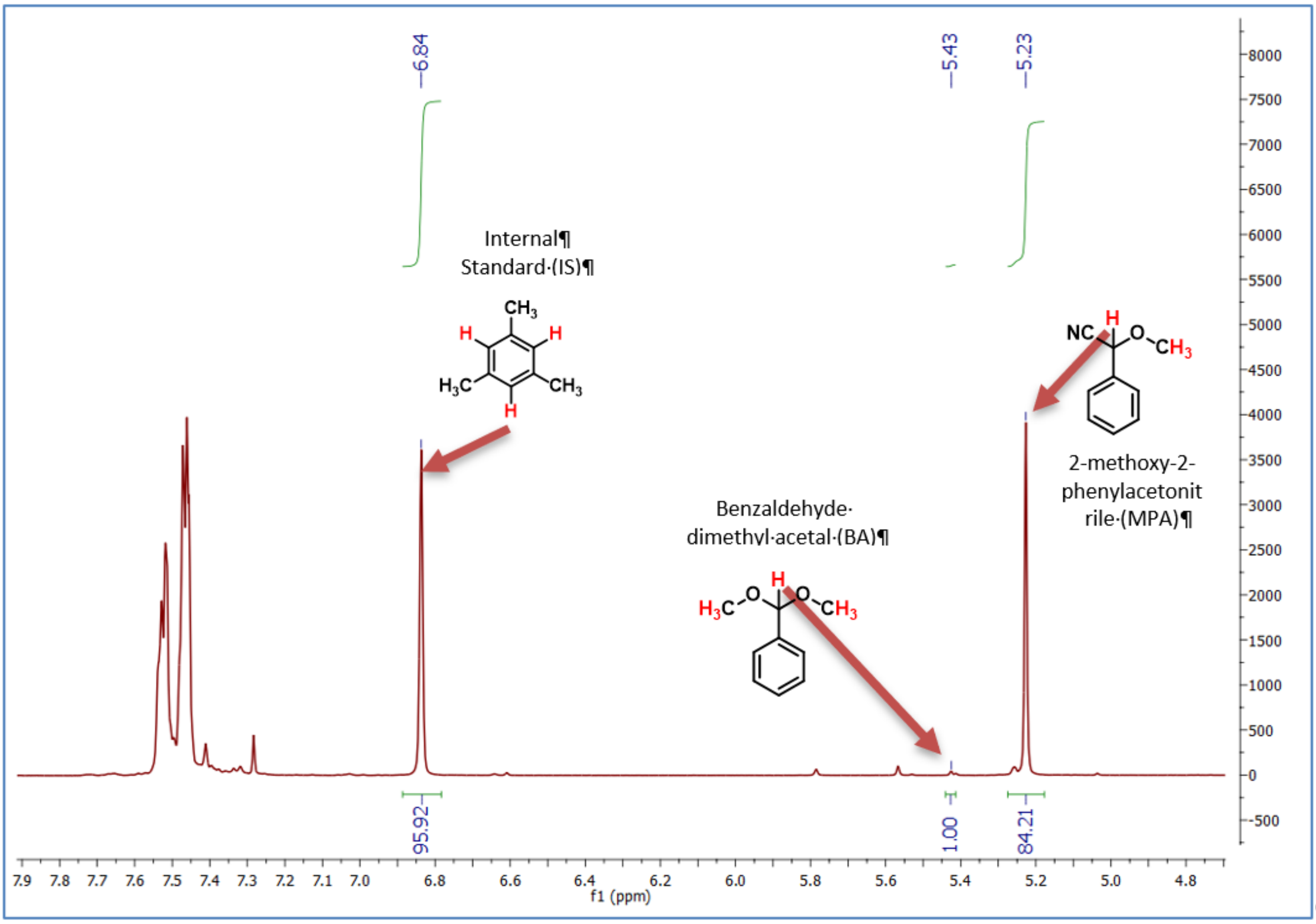

6.84
5.43
5.23
Internal¶
Standard·(IS)¶
Benzaldehyde·
dimethyl·acetal·(BA)¶
2-methoxy-2-
phenylacetonit
rile·(MPA)¶
95.92
1.00
84.21
f1 (ppm)

(A)

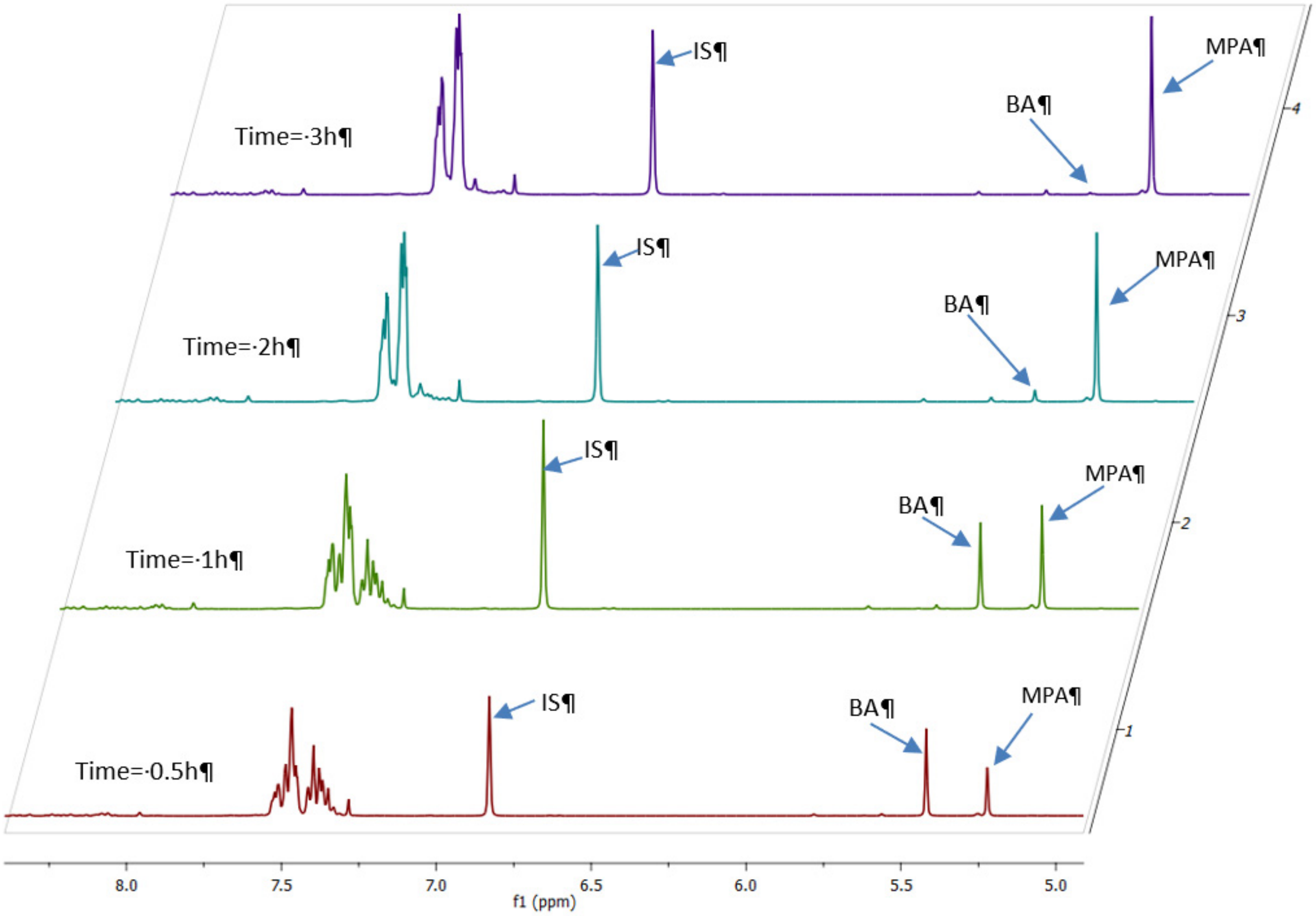

(B)

Figure S6. (A) ^{1}H-NMR spectra of the crude product of the solvent-free cyanation of benzaldehyde dimethyl acetal with CP **1** as catalyst in $CDCl_3$ (entries 1 and 2, Table 1) (The protons are considered in the integrations are indicated in red colour). (B) Time depended (0.5h to 3h) ^{1}H-NMR spectrum for the cyanation of benzaldehyde dimethyl acetal with catalyst CP **1**.

Reaction yield calculation details from NMR:

In order to calculate the % of yield from ^{1}H-NMR spectroscopy, at first, the molar amount of product (P) was calculated from the molar amount of internal standard (IS) and the molar ratio, where the following formula has been utilized to carry out all the calculations.

The molar ratio of the compound, $r_{P/IS}$ = (integral$_P$/N_P)/(integral$_{IS}$/N_{IS}) where N = number of nuclei present for the corresponding peaks.

The molar amount of product (P), $n_P = n_{IS} \times r_{P/IS}$

The NMR yield (in %) = (n_P x 100)/(theoretical yield of analyte)

The molar ratio of the compound, $r_{P/IS}$ = (84.21/1)/(95.92/3) = 2.63

The molar amount of internal standard (IS), n_{IS} : 0.18 mmol (25 µl)

The molar amount of product (P), $n_P = n_{IS} \times r_{P/IS}$ = 2.63 X 0.18 = 0.473 mmol

The NMR yield (in %) = (n_P x 100)/(theoretical yield of analyte) = (0.473X 100)/ 0.5 = 94.8%

Table S1. A comparison of catalytic activity of various catalysts in the cyanation of benzaldehyde dimethyl acetal reactions.

Entry	Catalyst	Cyanating agent	Time (h)	Temp (°C)	Yield (%)	Ref
1	$BiBr_3$	TMSCN	1	RT	89	23
2	MgI_2 etherate	TMSCN	36	RT	53	21
3	Tetracyanoethylene	TMSCN	5	Reflux	72	34
4	TESOTf, 2,2′-bipyridine	TMSCN	5	0	98	35
5	$TiCl_4$	*t*-butyl isocyanide	3	-70	96	36
6	$SiCl_3OTf$	Cyanoamine	0.5	0	95	32
7	$[Cd_4(L1)_4(DMF)_6]_n.3n(DMF)$	TMSCN	4	80	96	24
8	$[Cd_2(L2)_2(DMF)_3]_n.2n(DMF)$	TMSCN	4	80	94	24
9	CP **1**	TMSCN	3	70	95	Our work
10	CP **2**	TMSCN	3	70	84	Our work

TESOTf = triethylsilyl trifluoromethanesulfonate; L1 = 5-{(anthracen-9-ylmethyl)amino}isophthalate; L2 = 5-{(pyren-1-ylmethyl)amino}isophthalate.

2. Crystal structure determinations

X-ray quality single crystals of the compounds were immersed in cryo-oil, mounted in a nylon loop and measured at room temperature. Intensity data were collected using a Bruker APEX-II PHOTON 100 diffractometer with graphite monochromated Mo-Kα (λ 0.71069) radiation. Data were collected using phi and omega scans of 0.5° per frame and a full sphere of data was obtained. Cell parameters were retrieved using Bruker SMART [1] software and refined using Bruker SAINT [1] on all the observed reflections. Absorption corrections were applied using SADABS [2]. Structures were solved by direct methods by using the SHELXS-2014 package [3] and refined with SHELXL-2014/6 [3]. Calculations were performed using the WinGX System-Version 2014.1 [4]. The hydrogen atoms attached to carbon and nitrogen atoms were inserted at geometrically calculated positions and included in the refinement using the riding-model approximation; U_{iso}(H) were defined as 1.2U_{eq} of the parent atoms for phenyl and 1.5U_{eq} of the parent atoms for the methyl groups and nitrogen atoms. Least square refinements with anisotropic thermal motion parameters for all the non-hydrogen atoms and isotropic ones for the remaining atoms were employed. In CP **1** one coordinated DMF molecule is disordered over two orientations and were refined to a ratio of 52% and 48% with the use of PART instruction. Moreover CP **1** contained disordered water molecules which could not be modelled reliably. PLATON/SQUEEZE[43] was used to correct the data and only 12 electrons per unit cell worth of scattering which suggest the presence of one water molecule per unit cell. Crystallographic data are summarized in Table S1 (Supplementary Information file) and selected bond distances and angles are presented in Table S2. CCDC 2130710-2130711 contain the supplementary crystallographic data for this paper. These data can be obtained free of charge from The Cambridge Crystallographic Data Centre *via* www.ccdc.cam.ac.uk/data_request/cif (accessed on 1 December 2022).

Table S1. Crystal data and structure refinement details for compounds 1-2.

Identification name	1	2
Formula	$C_{29}H_{25}CdN_3O_6$	C26 H28 Cd N2 O8
M.W. / g·mol^{-1}	623.92	608.90
Crystal System	Triclinic	Monoclinic
Space Group	P-1	P 21
Temperature / K	296	296
Wavelength / Å	0.71073	0.71073
a / Å	8.0399(5)	10.1286(12)
b / Å	10.2245(6)	7.1032(8)
c / Å	16.2601(11)	17.274(2)
α / °	100.193(2)	90
β / °	102.525(3)	96.631(5)
γ / °	91.184(2)	90
V / Å^3	1281.81(14)	1234.5(2)
Z	2	2
Density / Mgm^{-3}	1.617	1.638
Abs. Coeff. / mm^{-1}	0.902	0.939
F(000)	632	620
Refl. collected	16617	8118
Refl. unique	4703	4387
Max. 2θ/°	25.391	25.374
Ranges (h, k, l)	-9<= h <=9<br>-12 <= k <=12<br>-19<= l <= 19	-12<= h <=11<br>-8 <= k <=8<br>-20<= l <= 15
Complete to 2θ (%)	99.3	99.7
Refl. with I > 2σ(I)	4311	2817
Data/Restraints/Parameters	4703/0/354	4387/130/298
Goof (F^2)	1.033	1.060
R1 [I > 2s(I)]	0.0391	0.0819
wR2 [I > 2s(I)]	0.1011	0.2094
R1 [all data]	0.0427	0.1455
wR2 [all data]	0.1051	0.2656

Table S2. Hydrogen bond geometry (Å, °) in compounds 1-2.

Compound	D-H···A	D···H (Å)	H···A (Å)	D···A (Å)	<D–H···A(°)
**1**	N1-H1N···O1	0.86	2.19	2.967(4)	151.0
	N2-H2N···O4	0.86	2.08	2.880(4)	155.4
	C25-H25···O6	0.93	2.63	3.338(5)	133.0
	C26-H26B···O3	0.96	2.38	3.301(5)	160.2
**2**	O3-H3A···O5	0.84	2.35	3.04(3)	139.4
	O3-H3B···O4	0.84	2.17	3.00(3)	168.3
	O4-H4A···O6	0.91	1.96	2.88(3)	176.8
	O4-H4B···O1S	0.94	1.76	2.70(3)	178.7
	O1S-H1A···O1	0.89	1.92	2.81(3)	179.2
	O1S-H1B···O1	0.91	1.86	2.77(2)	179.3

Table S3. Selected bond distances (Å) and angles (°) for compounds 1-2.

1	Cd1-O1 2.237(2), Cd1-O2 2.257(2), Cd1-O3 2.367(2), Cd1-O4 2.358(2), Cd1-O5 2.259(3), Cd1-O6 2.371(3).<br><O1-Cd1-O2 115.51(9), <O1-Cd1-O5 100.20(10), <O2-Cd1-O5 90.19(11), <O1-Cd1-O4 144.44(8), <O2-Cd1-O4 98.73(9), <O5-Cd1-O4 88.47(9), <O1-Cd1-O3 89.22(8), <O2-Cd1-O3 153.86(9), <O5-Cd1-O3 93.73(10), <O4-Cd1-O3 55.63(8), <O1-Cd1-O6 88.14(10), <O2-Cd1-O6 83.11(11), <O5-Cd1-O6 171.00(10), <O4-Cd1-O6 86.63(9), <O3-Cd1-O6 89.73(10).
**2**	Cd1-O1 2.665(2), Cd1-O2 2.253(11), Cd1-O3 2.26(2), Cd1-O4 2.19(3), Cd1-O5 2.354(14), Cd1-O6 2.475(13), Cd1-O7 2.313(12).<br><O4-Cd1-O2 89.7(9), <O4-Cd1-O3 161.6(11), <O2-Cd1-O3 94.3(9), <O4-Cd1-O7 109.2(11), <O2-Cd1-O7 84.6(4), <O3-Cd1-O7 89.0(10), <O4-Cd1-O5 77.9(13), <O2-Cd1-O5 139.9(4), <O3-Cd1- O5 87.6(13), <O7-Cd1-O5 135.5(4), <O4-Cd1-O6 100.1(9), <O2-Cd1-O6 138.9(4), <O3-Cd1-O6 88.7(9), <O7-Cd1-O6 54.4(4), <O5-Cd1-O6 81.1(5).

References

1. 1. Bruker, *APEX2*. Bruker AXS Inc., Madison, Wisconsin, USA, **2012**.
2. 2. Sheldrick, G.M. *SADABS. Program for Empirical Absorption Correction*. University of Gottingen, Germany, **1996**.
3. 3. Sheldrick, G.M. *Acta Crystallogr*. Crystal structure refinement with SHELXL. **2015**, *C71*, 3–8.
4. 4. Farrugia, L.J.J. *Appl. Crystallogr*. WinGX and ORTEP for Windows: an update. **2012**, *45*, 849–854.
